# An environmental psychology model of sustainability in sport: the roles of sustainability consciousness, environmental awareness, and personal social responsibility

**DOI:** 10.3389/fpsyg.2026.1933846

**Published:** 2026-09-03

**Authors:** Barış Karaoğlu, Oğulcan Usuflu, Harun Genç, Bilgehan Pepe, Berat Koçyiğit, Mehmet Behzat Turan, İbrahim Dalbudak, Osman Pepe

**Affiliations:** 1 Faculty of Sports Sciences, Bingol University, Bingöl, Türkiye; 2 Department of Management and Organization, Vocational School, Istanbul Rumeli University, Istanbul, Türkiye; 3 Faculty of Sport Sciences, Istanbul Gelisim University, Istanbul, Türkiye; 4 Faculty of Sports Sciences, Süleyman Demirel University, Isparta, Türkiye; 5 Faculty of Sports Sciences, Erciyes University, Kayseri, Türkiye; 6 Faculty of Sports Sciences, Uşak University, Uşak, Türkiye

**Keywords:** environmental awareness (EA), personal social responsibility, serial mediation models, sport sciences, sustainability consciousness, sustainability in sport

## Abstract

**Background:**

Understanding the psychological processes associated with sustainable behavior has become a major focus of environmental psychology. Although sustainability consciousness is generally associated with environmentally responsible behavior, the psychological processes underlying this association in sport remain insufficiently understood, particularly among sport science students. Therefore, the present study examined the serial mediating roles of environmental awareness and personal social responsibility in the association between sustainability consciousness and sustainability in sport.

**Methods:**

A cross-sectional correlational design was employed. The study included 521 undergraduate students enrolled in Faculties of Sport Sciences at six universities in Türkiye during the 2025–2026 academic year. Participants completed the sustainability consciousness scale, environmental awareness scale, personal social responsibility scale, and sustainability in sport scale. Serial mediation analysis was performed using Hayes’ PROCESS Macro (Model 6) with 5,000 bootstrap resamples. Prior to hypothesis testing, the assumptions required for regression analyses, including normality, multicollinearity, and the absence of influential cases, were examined and found to be satisfactory.

**Results:**

Sustainability consciousness was positively associated with environmental awareness, personal social responsibility, and sustainability in sport. Multiple regression analyses indicated that sustainability consciousness was positively associated with sustainability in sport. The serial mediation analysis further showed that sustainability consciousness was positively associated with environmental awareness and personal social responsibility, environmental awareness was positively associated with personal social responsibility, and both environmental awareness and personal social responsibility were positively associated with sustainability in sport. The direct association between sustainability consciousness and sustainability in sport remained statistically significant after accounting for the proposed mediators, suggesting partial mediation. Bootstrap analyses further indicated statistically significant indirect associations involving environmental awareness, personal social responsibility, and the sequential pathway including both constructs, as none of the 95% bootstrap confidence intervals included zero. These findings support the proposed serial mediation model as a theoretically informed pattern of statistical associations rather than evidence of causal mediation.

**Conclusion:**

The findings suggest that sustainability consciousness, environmental awareness, personal social responsibility, and sustainability in sport are statistically associated in a manner consistent with the proposed environmental psychology framework. By examining these constructs within a serial mediation model, the present study contributes to the environmental psychology literature by providing a theoretically grounded perspective on the statistical associations underlying sustainability-related orientations within the sport context. The findings also have practical implications for the development of sustainability education and environmental responsibility initiatives within Faculties of Sport Sciences and provide a foundation for future longitudinal and experimental research examining these associations.

## 1 Introduction

In recent decades, accelerating climate change, biodiversity loss, environmental degradation, and the rapid depletion of natural resources have transformed sustainability into one of the most pressing global challenges. These environmental crises have shifted sustainability from being solely a policy objective to becoming a multidisciplinary scientific field concerned with understanding and promoting behavioral change toward more sustainable societies (United Nations, 2019). In its broadest sense, sustainability refers to a development model that satisfies present needs without compromising the ability of future generations to meet their own needs [World Commission on Environment and Development (WCED), 1987]. Contemporary sustainability research conceptualizes sustainability as an integrated framework comprising environmental, social, and economic dimensions rather than focusing exclusively on environmental protection. Consequently, sustainability has become an important research area across numerous disciplines, including environmental sciences, psychology, education, economics, business, and sport.

Among these disciplines, environmental psychology has become one of the leading scientific fields for understanding why individuals engage in or fail to engage in environmentally responsible behaviors. Rather than viewing sustainability simply as an issue of environmental knowledge, environmental psychology proposes that sustainable behavior emerges through complex interactions among cognitive processes, environmental perceptions, emotions, personal values, moral evaluations, and social norms (Steg and de Groot, 2019). Accordingly, recent research increasingly emphasizes that promoting sustainability requires a better understanding of the psychological processes underlying the association between environmental knowledge and environmentally responsible behavior. From this perspective, sustainable behavior is viewed not merely as an environmental outcome but also as a psychological phenomenon associated with multiple cognitive and motivational factors.

One of the central debates within environmental psychology concerns the well-documented knowledge–behavior gap, which suggests that possessing sustainability-related knowledge does not necessarily lead individuals to adopt environmentally responsible behaviors. Although individuals may demonstrate high levels of environmental knowledge and awareness, these characteristics alone often fail to predict actual sustainable actions (Kollmuss and Agyeman, 2002). Previous studies indicate that behavioral change depends on additional psychological mechanisms such as environmental concern, environmental awareness, perceived responsibility, moral norms, and social values (Steg and Vlek, 2009). Consequently, identifying the psychological processes that bridge sustainability consciousness and sustainable behavior has become one of the primary objectives of contemporary environmental psychology research.

Within this theoretical perspective, sustainability consciousness represents a multidimensional psychological construct encompassing individuals’ knowledge, awareness, values, and attitudes regarding environmental, social, and economic sustainability (Gericke et al., 2019). Individuals with higher sustainability consciousness are generally more likely to support environmentally responsible decisions and adopt more sustainable lifestyles (Kollmuss and Agyeman, 2002; Li et al., 2019). Nevertheless, sustainability consciousness alone rarely guarantees sustainable behavior because behavioral adoption depends on how cognitive awareness is associated with psychological motivation and perceived responsibility. Therefore, contemporary environmental psychology increasingly focuses on identifying intermediate psychological processes that may help explain the association between sustainability consciousness and sustainable behavior.

One of these processes is environmental awareness, which reflects not only individuals’ knowledge of environmental issues but also their ability to cognitively evaluate environmental consequences, recognize ecological problems, and develop sensitivity toward environmental degradation (Bamberg and Möser, 2007). Environmental awareness represents a fundamental psychological construct associated with pro-environmental behavior because individuals who perceive environmental problems more clearly are generally more willing to support environmentally responsible actions (Steg and Vlek, 2009). Furthermore, environmental awareness has been closely associated with the development of Environmental Identity, referring to the extent to which individuals incorporate nature into their self-concept. Previous environmental psychology research suggests that stronger Environmental Identities are associated with higher levels of environmentally responsible behavior because individuals often behave in ways that are consistent with identities they perceive as central to themselves (Clayton, 2021). Although environmental identity was not directly examined in the present study, this perspective provides a useful theoretical framework for understanding the association between environmental awareness and sustainable behavior.

Another important psychological construct associated with sustainable behavior is personal social responsibility. From an environmental psychology perspective, personal social responsibility reflects individuals’ perceived obligation to contribute to solving environmental and societal problems through their own actions. This construct can also be interpreted within Norm Activation Theory, which proposes that environmentally responsible behavior emerges when individuals recognize environmental consequences and simultaneously perceive themselves as personally responsible for addressing those consequences (Schwartz, 1977). Accordingly, greater environmental awareness may be associated with stronger perceptions of personal social responsibility, which, in turn, may be associated with sustainability-oriented behavioral choices. Previous studies consistently suggest that individuals possessing stronger senses of social and moral responsibility tend to report higher levels of both prosocial and environmentally responsible behavior (Steg and Vlek, 2009).

These psychological processes are particularly relevant within the field of sport. The sport sector represents a socially influential context because of its broad societal reach and its potential to promote sustainability-related values, shape lifestyles, influence consumption patterns, and contribute to the formation of social norms (McCullough and Kellison, 2018; McCullough et al., 2020). At the same time, sport organizations, facilities, and major sporting events generate considerable environmental impacts through energy consumption, transportation-related emissions, resource utilization, and waste production (Trendafilova et al., 2013). Consequently, sustainability in sport extends beyond reducing environmental impacts at the organizational level; it also requires promoting sustainable behaviors among athletes, students, coaches, administrators, and future sport professionals (Orr et al., 2020). From an environmental psychology perspective, sport provides an important social context for examining the statistical associations among sustainability-related psychological processes and environmentally responsible behavior.

Despite the growing literature on sustainability in sport, several important research gaps remain. First, relatively few studies have examined sustainability in sport from an environmental psychology perspective. Second, although sustainability consciousness has frequently been associated with environmentally responsible behavior, the psychological processes underlying this association remain insufficiently understood. Third, serial mediation models simultaneously integrating sustainability consciousness, environmental awareness, and personal social responsibility have rarely been examined among sport sciences students. Addressing these gaps may contribute not only to sport sustainability research but also to the broader environmental psychology literature by providing a more comprehensive understanding of the statistical associations among sustainability consciousness, environmental awareness, personal social responsibility, and sustainability in sport.

Accordingly, the present study investigates the association between sustainability consciousness and sustainability in sport among sport sciences students by examining the serial mediating roles of environmental awareness and personal social responsibility. Beyond examining direct associations, the proposed model provides a theoretically informed framework for understanding the statistical associations among sustainability consciousness, environmental awareness, personal social responsibility, and sustainability in sport. In doing so, the study contributes to environmental psychology by extending current knowledge regarding the statistical associations among key psychological constructs associated with sustainability in sport while simultaneously providing empirical evidence relevant to sustainability education within higher education and sport sciences.

The findings also contribute to the implementation of the United Nations Sustainable Development Goals (SDGs), particularly SDG 4 (Quality Education), SDG 12 (Responsible Consumption and Production), and SDG 13 (Climate Action). By examining the psychological correlates of sustainability in sport, the study provides practical implications for developing sustainability-oriented educational programs, environmental awareness interventions, and psychologically informed behavioral strategies within sport education. More importantly, the proposed serial mediation model extends the environmental psychology literature by providing a theoretically grounded framework for understanding the statistical associations among sustainability consciousness, environmental awareness, personal social responsibility, and sustainability in sport within the sport context.

### 1.1 Sustainability consciousness

Sustainability consciousness is a broad multidimensional construct encompassing knowledge, attitudes, and self-reported behavioral orientations across the environmental, social, and economic dimensions of sustainable development (Gericke et al., 2019). Rather than reflecting the simple acquisition of environmental information, it refers to individuals’ understanding of sustainability-related issues, recognition of their long-term consequences, and integration of sustainability principles into decision-making (Li et al., 2019). From an environmental psychology perspective, sustainability consciousness therefore represents a broad sustainability-related orientation associated with how individuals interpret environmental challenges and evaluate their own role in sustainable development.

However, sustainability consciousness alone may be insufficient to account for sustainability-related outcomes. Although individuals with higher levels of sustainability consciousness tend to report more sustainable consumption, energy conservation, waste reduction, and environmentally responsible lifestyle choices, the translation of knowledge and attitudes into behavior is not always direct (Kollmuss and Agyeman, 2002; Li et al., 2019). This knowledge–behavior gap suggests that additional psychological constructs may be associated with the relationship between sustainability consciousness and sustainability-related outcomes.

Within the present framework, environmental awareness and personal social responsibility are considered theoretically relevant constructs in this regard. Sustainability consciousness reflects a broad sustainability-related orientation, whereas environmental awareness concerns the more specific recognition and appraisal of environmental problems and their consequences (Bamberg and Möser, 2007). Greater sustainability consciousness may therefore be associated with stronger environmental awareness, which may in turn be associated with stronger perceptions of personal social responsibility toward environmental and societal well-being (Gifford and Nilsson, 2014; Arnocky et al., 2007). Accordingly, sustainability consciousness is positioned as the broad starting construct in the proposed statistical framework linking environmental awareness, personal social responsibility, and sustainability in sport.

Sustainability consciousness may also be strengthened through educational experiences. Education for sustainable development emphasizes that sustainability education should extend beyond knowledge transmission by fostering critical thinking, environmental values, ethical responsibility, and competencies related to sustainable action (Wals and Benavot, 2017). This perspective is especially relevant for sport sciences students, who are likely to assume future professional roles in coaching, physical education, sport management, and recreation and may contribute to sustainability-related practices within sport settings.

### 1.2 Environmental awareness

Environmental awareness is a central construct in environmental psychology that reflects how individuals recognize, interpret, and appraise environmental problems and their consequences. Unlike the broader multidimensional orientation represented by sustainability consciousness, environmental awareness is more specifically concerned with the salience, perceived relevance, and appraisal of environmental issues (Bamberg and Möser, 2007; Steg and Vlek, 2009). Accordingly, it is conceptualized in the present study as a more focused environmental appraisal construct that is theoretically distinct from sustainability consciousness.

Environmental awareness encompasses more than factual environmental knowledge. It involves cognitive and affective evaluations of environmental issues, including environmental concern and the perceived personal relevance of ecological problems (Dunlap et al., 2000). Individuals with higher levels of environmental awareness generally report stronger environmentally responsible orientations and greater support for sustainable practices such as recycling, energy conservation, waste reduction, and sustainable consumption (Bamberg and Möser, 2007). However, awareness alone may be insufficient to account for sustainability-related outcomes, indicating that additional normative and motivational constructs should also be considered (Kollmuss and Agyeman, 2002; Steg and Vlek, 2009).

Within the proposed framework, environmental awareness is positioned between sustainability consciousness and personal social responsibility. Higher levels of sustainability consciousness may be associated with stronger recognition and appraisal of environmental problems, while greater environmental awareness may in turn be associated with stronger perceptions of personal responsibility for environmental and societal well-being (Gericke et al., 2019; Gifford and Nilsson, 2014). Accordingly, environmental awareness is treated as the first more specific psychological construct in the proposed statistical ordering.

This construct is particularly relevant within sport sciences because sport facilities, organizations, events, and outdoor physical activities are closely associated with environmental resource use and sustainability-related practices (McCullough and Kellison, 2018; Mallen and Chard, 2012). Sport sciences students, as future coaches, physical education teachers, sport managers, and recreation specialists, may therefore represent an important population for examining environmental awareness in relation to sustainability-oriented practices within sport settings.

### 1.3 Personal social responsibility

Personal social responsibility is a socio-psychological construct reflecting individuals’ perceived obligation to consider the consequences of their actions for society and the natural environment. From an environmental psychology perspective, it represents an internalized normative orientation associated with individuals’ perceived responsibility to contribute to environmental and societal well-being (Schwartz, 1977; Stern, 2000; Kollmuss and Agyeman, 2002). In contrast to sustainability consciousness, which reflects a broad sustainability-related orientation, and environmental awareness, which concerns the recognition and appraisal of environmental problems, personal social responsibility primarily reflects perceived ethical and social obligation. Accordingly, it is conceptualized as a distinct normative construct within the proposed framework.

Norm Activation Theory provides a theoretical rationale for positioning personal social responsibility after environmental awareness. The theory proposes that awareness of environmental consequences is associated with the attribution or acceptance of personal responsibility for addressing those consequences (Schwartz, 1977). In the present study, personal social responsibility is therefore conceptually related to this normative process, although it is not treated as a direct operational equivalent of personal norms. This distinction supports the proposed statistical ordering from environmental awareness to personal social responsibility.

Previous research has reported positive associations between personal social responsibility and environmentally responsible practices, including sustainable consumption, recycling, conservation, and resource protection (Steg and Vlek, 2009). Greater environmental awareness has likewise been associated with stronger perceptions of responsibility toward environmental and societal problems (Gifford and Nilsson, 2014). These findings support the view that personal social responsibility may represent an important normative construct statistically associated with sustainability-related outcomes.

This construct is particularly relevant within sport because sport settings involve continuous interaction with organizations, communities, resources, and the natural environment. Future coaches, physical education teachers, sport managers, and recreation specialists may contribute to sustainability-oriented practices through resource use, facility management, event organization, and environmental education. Accordingly, personal social responsibility is positioned as the second intermediate construct in the proposed model and is examined as statistically associated with sustainability in sport rather than as evidence of a temporally or causally subsequent psychological stage.

### 1.4 Sustainability in sport

Sustainability in sport has become an increasingly important research area because sport organizations, facilities, and events generate environmental, social, and economic impacts through resource use, energy consumption, transportation, waste production, and infrastructure development (Mallen and Chard, 2012; Trendafilova et al., 2013). Accordingly, sustainability in sport has developed into a multidimensional concept encompassing environmental, social, economic, organizational, and individual dimensions.

In the present study, sustainability in sport is conceptualized as the domain-specific outcome construct within the proposed framework. It represents a multidimensional sustainability orientation situated within the sport context and includes individual, social, participation-related, economic, organizational, and environmental dimensions. Therefore, higher scores should be interpreted as indicating a stronger overall sustainability orientation within sport settings rather than as direct evidence of objectively observed sustainable behavior.

Although sustainability in sport has traditionally been examined from organizational and managerial perspectives, including facility management, resource efficiency, sustainable event organization, and environmental policy, recent research has increasingly emphasized the importance of individual-level sustainability orientations among athletes, coaches, spectators, volunteers, managers, and students (McCullough and Kellison, 2018; McCullough et al., 2020). From an environmental psychology perspective, these orientations may be associated with cognitive appraisals, environmental perceptions, personal values, and perceived responsibility (Steg and de Groot, 2019).

Within the proposed model, sustainability consciousness, environmental awareness, and personal social responsibility are treated as conceptually related psychological constructs statistically associated with sustainability in sport. This perspective is particularly relevant for sport sciences students because they are likely to assume future professional roles in coaching, physical education, sport management, and recreation and may contribute to sustainability-related practices within sport organizations and communities.

### 1.5 The present study

Despite growing interest in sustainability in sport, relatively few studies have examined this construct from an environmental psychology perspective. Existing research has focused largely on organizational sustainability strategies, environmental management, and policy-related issues, whereas the statistical associations among sustainability consciousness, environmental awareness, personal social responsibility, and sustainability in sport remain less understood. In particular, these constructs have rarely been examined together within a serial mediation framework among sport sciences students.

To address this gap, the present study examines a serial mediation model in which environmental awareness and personal social responsibility are specified as sequential intermediate constructs in the association between sustainability consciousness and sustainability in sport. The proposed model is primarily informed by Value-Belief-Norm (VBN) Theory (Stern, 2000), with complementary support from Norm Activation Theory (Schwartz, 1977). VBN Theory provides the broader rationale for linking sustainability-related cognitions and environmental appraisals with normative responsibility and sustainability-related outcomes, whereas Norm Activation Theory provides more specific support for the association between environmental awareness and personal social responsibility.

Within this framework, sustainability consciousness is positioned as a broad sustainability-related orientation, environmental awareness as a more specific environmental appraisal construct, personal social responsibility as a normative construct, and sustainability in sport as the domain-specific outcome. Accordingly, the proposed statistical ordering is sustainability consciousness → environmental awareness → personal social responsibility → sustainability in sport. Given the cross-sectional design, this ordering is intended to represent theoretically specified statistical associations rather than temporal or causal progression.

The study is expected to contribute to environmental psychology and sustainability research by providing a more integrated account of these constructs within the sport context and by offering a basis for future longitudinal and experimental research.

### 1.6 Theoretical framework, mediation pathway, and theoretical contribution

The present study is primarily grounded in the Value-Belief-Norm (VBN) Theory (Stern, 2000), with complementary support from Norm Activation Theory (Schwartz, 1977). VBN Theory provides the broader conceptual rationale for linking sustainability-related cognitions and environmental appraisals with normative responsibility and sustainability-related outcomes, whereas Norm Activation Theory provides more specific support for the association between environmental awareness and personal social responsibility. The study constructs are not treated as direct operational equivalents of the original theoretical components; rather, these perspectives are used selectively to justify the proposed statistical ordering. Accordingly, the research question and hypotheses were theoretically derived from these frameworks rather than formulated solely on an exploratory or researcher-driven basis.

Within this framework, sustainability consciousness is conceptualized as a broad multidimensional sustainability-related orientation, environmental awareness as a more specific environmental appraisal construct, personal social responsibility as a normative construct reflecting perceived ethical and social obligation, and sustainability in sport as a multidimensional, domain-specific sustainability orientation within the sport context. Although these constructs are theoretically related, their differing conceptual referents support their treatment as distinct constructs. Some content overlap between sustainability consciousness and sustainability in sport is nevertheless plausible because both include attitudinal and self-reported behavioral elements; this issue was therefore considered in the measurement evaluation and interpretation of the findings.

The proposed statistical ordering is sustainability consciousness → environmental awareness → personal social responsibility → sustainability in sport. This ordering reflects the theoretical distinction between a broad sustainability-related orientation, environmental appraisal, normative responsibility, and a domain-specific sustainability outcome. Given the cross-sectional design, the proposed sequence should be interpreted as a theoretically specified pattern of statistical associations rather than evidence of temporal, developmental, or causal progression.

By integrating these constructs within a single serial mediation framework, the study extends environmental psychology research into the sport context and provides a parsimonious basis for examining their direct and indirect statistical associations.

Accordingly, the main research question guiding the present study is: To what extent is sustainability consciousness statistically associated with sustainability in sport among sport sciences students, and to what extent are environmental awareness and personal social responsibility involved in this association, both independently and through the theoretically specified sequential pathway from sustainability consciousness to environmental awareness, from environmental awareness to personal social responsibility, and subsequently to sustainability in sport? Given the cross-sectional design, this research question concerns theoretically specified direct and indirect statistical associations and does not imply temporal or causal relationships among the constructs.

Based on this theoretical framework, the following hypotheses were developed:

*H1*. Sustainability consciousness is positively associated with environmental awareness.

*H2*. Sustainability consciousness is positively associated with personal social responsibility.

*H3*. Environmental awareness is positively associated with personal social responsibility.

*H4*. Sustainability consciousness remains positively associated with sustainability in sport after accounting for environmental awareness and personal social responsibility.

*H5*. Environmental awareness is positively associated with sustainability in sport after accounting for sustainability consciousness and personal social responsibility.

*H6*. Personal social responsibility is positively associated with sustainability in sport after accounting for sustainability consciousness and environmental awareness.

*H7*. Sustainability consciousness is indirectly associated with sustainability in sport through environmental awareness.

*H8*. Sustainability consciousness is indirectly associated with sustainability in sport through personal social responsibility.

*H9*. Sustainability consciousness is indirectly associated with sustainability in sport through the sequential statistical pathway involving environmental awareness and personal social responsibility.

## 2 Materials and methods

### 2.1 Study design

This study employed a cross-sectional correlational research design to examine the statistical associations among sustainability consciousness, environmental awareness, personal social responsibility, and sustainability in sport. Cross-sectional correlational designs are appropriate for investigating the magnitude and direction of relationships among variables without manipulating study conditions or permitting causal inferences (Christensen et al., 2010; Karasar, 1999).

The primary aim of the study was to examine the association between Sustainability Consciousness and Sustainability in Sport and to evaluate the statistical indirect associations involving environmental awareness and personal social responsibility within a theoretically informed serial mediation framework.

Accordingly, sustainability consciousness was specified as the independent variable (X), sustainability in sport as the dependent variable (Y), environmental awareness as the first mediator (M1), and personal social responsibility as the second mediator (M2). The hypothesized serial mediation model was specified using Hayes' (2022) PROCESS Macro Model 6 to estimate the direct and indirect statistical associations among the study variables. Given the cross-sectional nature of the study, the mediation analyses were interpreted as tests of statistically significant indirect associations rather than evidence of causal mediation.

### 2.2 Determining sample size using Monte Carlo simulation

An *a priori* Monte Carlo simulation was used to determine the sample size required to achieve adequate statistical power for the hypothesized serial indirect association in the PROCESS Model 6 framework. The simulation was based on the proposed sequence of Sustainability Consciousness (X) → Environmental Awareness (M1) → Personal Social Responsibility (M2) → Sustainability in Sport (Y). The primary effect of interest was the serial indirect effect represented by the product of the X → M1, M1 → M2, and M2 → Y paths (a₁ × d₂₁ × b₂).

Based on the medium-effect assumptions commonly used in mediation power evaluations and the guidance of Fritz and MacKinnon (2007), standardized coefficients of approximately 0.30 were specified for each of the three component paths forming the serial indirect effect (a₁ = 0.30, d₂₁ = 0.30, and b₂ = 0.30). Accordingly, the assumed standardized serial indirect effect was approximately 0.027 (0.30 × 0.30 × 0.30). Statistical significance was evaluated using a two-sided *α* level of 0.05, corresponding to a 95% confidence level, and the target statistical power was set at 0.80.

For each candidate sample size, 2,000 Monte Carlo replications were generated under the prespecified population parameters. Statistical power was defined as the proportion of simulated samples in which the 95% confidence interval for the serial indirect effect excluded zero. Candidate sample sizes were evaluated iteratively until the estimated power reached or exceeded 0.80. Under these assumptions, a sample size of approximately 400 participants was estimated to provide at least 80% power for detecting the hypothesized serial indirect association. The final analytical sample consisted of 521 participants, exceeding the simulation-based minimum sample-size requirement.

### 2.3 Participants

The study was conducted during the 2025–2026 academic year among undergraduate students enrolled in the Faculties of Sport Sciences at six universities in Türkiye. The distribution of participants across the six participating universities was as follows: Bingöl University, *n* = 110 (21.1%); Samsun Ondokuz Mayıs University, *n* = 90 (17.3%); Fırat University, *n* = 85 (16.3%); Erciyes University, *n* = 80 (15.4%); Istanbul Rumeli University, *n* = 76 (14.6%); and Munzur University, *n* = 80 (15.4%). A total of 521 students from the departments of physical education and sport teaching, coaching education, sport management, and recreation participated in the study. Participants were recruited using a non-probability convenience sampling approach based on accessibility and voluntary participation across the participating universities. Accordingly, the sampling procedure was not intended to produce a statistically representative sample of all sport sciences students in Türkiye. The online questionnaire was distributed to 521 eligible students, all of whom completed and submitted the survey, resulting in a response rate of 100%. No reduction occurred between questionnaire submission and the final analytical sample; all 521 submitted questionnaires were complete, usable, and retained for analysis, corresponding to a retention rate of 100%.

The inclusion criteria were as follows: (a) being an active undergraduate student enrolled in one of the departments of physical education and sport teaching, coaching education, sport management, or recreation within a Faculty of Sport Sciences; (b) being 18 years of age or older; (c) having sufficient Turkish language proficiency to understand and complete the study questionnaires; (d) providing voluntary informed consent; and (e) completing all study measures. The predefined exclusion criteria included enrollment outside the Faculty of Sport Sciences, age below 18 years, absence of informed consent, incomplete questionnaire completion, duplicate submissions, or responses exhibiting apparent inconsistencies that rendered the questionnaire unusable. None of the submitted questionnaires met these exclusion criteria.

Prior to data collection, permission was obtained from the administrations of the participating universities. In collaboration with faculty members, an invitation describing the study and containing a link to the online questionnaire developed using Google Forms was distributed to eligible students. The first page of the questionnaire included detailed information regarding the study objectives, procedures, voluntary participation, confidentiality, and participants’ rights. Only individuals who provided electronic informed consent were able to access the questionnaire.

The study was approved by the relevant institutional ethics committee and conducted in accordance with the ethical principles of the Declaration of Helsinki. Prior to analysis, the submitted questionnaires were screened for eligibility, completeness, duplicate records, and apparent response-quality problems. No item-level missing data, duplicate submissions, incomplete questionnaires, or apparently unusable responses were identified. Consequently, no missing-data imputation, casewise deletion, or other statistical treatment of missing values was required. All 521 submitted questionnaires satisfied the eligibility and data-quality requirements and were retained for the final analyses, with no cases excluded from the analytical sample.

### 2.4 Measures

#### 2.4.1 Personal Information Form

A Personal Information Form was developed to collect demographic and background information about the participants. The form included seven variables: gender, age, year of study, academic department, grade point average (GPA), previous participation in sustainability-related education or training, and monthly income level.

Table 1 presents the demographic and educational characteristics of the participants. Of the 521 sport sciences students, 56.8% were male and 43.2% were female. Regarding age, 74.5% were between 18 and 22 years, 15.7% were between 23 and 27 years, and 9.8% were aged 28 years or older. With respect to academic year, 28.2% were second-year students, 26.3% were first-year students, 25.3% were third-year students, and 20.2% were fourth-year students. Regarding academic department, 30.1% were enrolled in Coaching Education, 28.6% in Recreation, 22.8% in Sport Management, and 18.4% in Physical Education and Sport Teaching. In terms of academic achievement, the majority of participants (61.6%) reported a grade point average (GPA) between 2.00 and 2.99, followed by 30.3% with a GPA between 3.00 and 4.00 and 8.1% with a GPA below 2.00. Finally, regarding sustainability education, 25.3% of the participants reported having received sustainability-related education, whereas 74.7% indicated that they had not.

**Table 1:** Descriptive statistics: frequency and percentage values.

Variables	Groups	*n*	%
Gender	Female	225	43.2
	Male	296	56.8
Age	18–22	388	74.5
	23–27	82	15.7
	28 and above	51	9.8
Class	First	137	26.3
	Second	147	28.2
	Third	132	25.3
	Fourth	105	20.2
Department	Physical education and sport	96	18.4
	Coaching education	157	30.1
	Sport management	119	22.8
	Recreation expertation	149	28.6
Academic achievement	0–1.99	42	8.1
	2.00–2.99	321	61.6
	3.00–4.00	158	30.3
Sustainability training	Yes	132	25.3
	No	389	74.7

#### 2.4.2 Sustainability consciousness scale

To assess participants’ sustainability consciousness, the sustainability consciousness questionnaire (SCQ), originally developed by Michalos et al. (2012) and subsequently revised by Gericke et al. (2019), was used. The Turkish adaptation of the questionnaire was performed by Yüksel and Yıldız (2019). The questionnaire consists of 50 items grouped into three dimensions assessing knowledge, attitudes, and behaviors related to the environmental, social, and economic dimensions of sustainable development. Responses are recorded on a five-point Likert scale ranging from 1 (*strongly disagree*) to 5 (*strongly agree*). In the original validation study, the questionnaire demonstrated excellent internal consistency, with Cronbach’s alpha coefficients exceeding 0.90. In the Turkish adaptation study, the overall reliability coefficient was reported as *α* = 0.88. A sample item is: *“Economic development is necessary for sustainable development.”*

#### 2.4.3 Environmental awareness scale

Participants’ environmental awareness was assessed using the environmental awareness scale developed by Çetin and Yalçınkaya (2018). The final version of the scale comprises 14 items distributed across four dimensions. Responses are rated on a five-point Likert-type scale ranging from 1 (*strongly disagree*) to 5 (*strongly agree*), with higher scores reflecting greater environmental awareness. In the original validation study, the Cronbach’s alpha coefficient was 0.792 for the total scale. The internal consistency coefficients for the four dimensions were 0.692, 0.694, 0.530, and 0.593, respectively. A sample item is: *“In my opinion*. *Every person should be informed about the environment.”*

#### 2.4.4 Personal social responsibility scale

To assess participants’ personal social responsibility, the personal social responsibility scale developed by López Davis et al. (2021) was used. The Turkish adaptation of the scale was performed by Kocar and Seki (2023). The scale consists of 19 items organized into five dimensions: philanthropic, environmental, ethical, legal, and economic responsibility. Responses are recorded on an 11-point Likert scale ranging from 0 (strongly disagree) to 10 (strongly agree). In the original validation study, the Cronbach’s alpha coefficients exceeded 0.79, and item factor loadings ranged from 0.56 to 0.86. A sample item is: “I collaborate with a non-governmental organization.”

#### 2.4.5 Sustainability scale in sport

To assess participants’ sustainability in sport, the sustainability in sport scale (SSS) developed by Koçak et al. (2015) was used. The scale consists of 35 items, including 23 positively worded items and 12 negatively worded items. Responses are recorded on a five-point Likert scale ranging from 1 (strongly disagree) to 5 (strongly agree), with negatively worded items reverse-coded prior to analysis. The scale comprises six dimensions: individual, social, participation, economic, organizational, and environmental. Accordingly, the scale should be interpreted as assessing a broad, multidimensional sustainability orientation within the sport context, encompassing individual, social, participatory, economic, organizational, and environmental dimensions, rather than directly observed sustainable behavior. In the original validation study, exploratory factor analysis supported the six-factor structure of the scale, and the overall Cronbach’s alpha coefficient was reported as *α* = 0.92. A sample item is: “Physical education and sports training enable individuals to be more sensitive to social events.”

### 2.5 Common method bias

Because all study variables were assessed using self-report measures administered within the same survey, the potential influence of common method variance was considered. As an initial diagnostic, Harman’s single-factor test was conducted by entering all measurement items into an unrotated exploratory factor analysis. The first unrotated factor accounted for 28.20% of the total variance. Although this value was below the commonly used 50% reference value, Harman’s single-factor test was treated only as an initial diagnostic and not as definitive evidence that common method variance was absent.

In addition, procedural remedies were implemented during data collection to reduce the potential influence of common method effects. Participation was anonymous and confidential, which was intended to reduce evaluation apprehension and socially desirable responding.

Complementary evidence was also considered from the measurement evaluation. The separately estimated confirmatory measurement models demonstrated acceptable to good fit, and the study constructs showed empirical distinctiveness according to the convergent- and discriminant-validity assessments reported in Section 2.6. In particular, all HTMT values were below the conservative 0.85 threshold. Although these findings do not constitute a direct test of common method variance, they provide complementary evidence that the study measures did not reflect a single undifferentiated construct.

Nevertheless, because all variables were self-reported and collected at the same time, common method variance cannot be completely excluded. Accordingly, the observed associations should be interpreted with appropriate caution.

### 2.6 Measurement evaluation

Prior to testing the hypothesized associations, confirmatory factor analyses were conducted to evaluate the measurement structure of the study instruments. Given the multidimensional structure of the scales, the theoretically specified factor structure of each instrument was examined separately. Model fit was evaluated using the chi-square statistic (*χ*^2^), the *χ*^2^/df ratio, Comparative Fit Index (CFI), Tucker–Lewis Index (TLI), Root Mean Square Error of Approximation (RMSEA), and Standardized Root Mean Square Residual (SRMR).

As shown in Table 2, the measurement models demonstrated acceptable to good overall fit. The χ^2^/df values ranged from 2.45 to 3.46, CFI values ranged from 0.949 to 0.962, TLI values ranged from 0.921 to 0.951, and SRMR values ranged from 0.038 to 0.054. RMSEA values were also within acceptable limits across the measurement models. Collectively, these findings provided support for the specified measurement structures of the study instruments.

**Table 2:** Fit indices of the individual measurement models.

Model	*χ*^2^/df	CFI	TLI	SRMR	RMSEA
Sustainability consciousness	2.84	0.958	0.940	0.046	0.054
Environmental awareness	2.67	0.962	0.951	0.038	0.056
Personal social responsibility	2.45	0.949	0.929	0.041	0.061
Sustainability in sport	3.46	0.953	0.921	0.054	0.068

Table 3 presents the reliability and convergent-validity statistics for the study constructs. Cronbach’s alpha coefficients ranged from 0.733 to 0.960, while composite reliability values ranged from 0.796 to 0.935, exceeding the recommended threshold of 0.70. The AVE values ranged from 0.502 to 0.742 and were therefore at or above the recommended 0.50 criterion, supporting convergent validity. In addition, the AVE for each construct exceeded its corresponding maximum shared variance (MSV), providing complementary evidence in support of construct distinctiveness despite the theoretical relatedness among the study constructs. MaxR(H) values ranged from 0.858 to 0.946, further supporting construct reliability.

**Table 3:** Reliability and convergent validity assessment of the study constructs.

Construct	*α*	CR	AVE	MSV	MaxR(H)
Sustainability consciousness	0.954	0.893	0.738	0.128	0.939
Environmental awareness	0.733	0.796	0.502	0.466	0.858
Personal social responsibility	0.960	0.935	0.742	0.412	0.946
Sustainability in sport	0.936	0.934	0.703	0.466	0.937

*α*, Cronbach’s alpha; CR, Composite Reliability; AVE, Average Variance Extracted; MSV, Maximum Shared Variance; MaxR(H), Maximum Reliability H. Recommended thresholds are *α* ≥ 0.70, CR ≥ 0.70, AVE ≥ 0.50, and AVE > MSV (Fornell and Larcker, 1981; Hair et al., 2021).

#### 2.6.1 Discriminant validity assessment

Discriminant validity was examined by comparing the square root of the Average Variance Extracted (AVE) for each construct with the observed correlations among the corresponding composite scores. This comparison was considered as complementary evidence regarding construct distinctiveness, with reference to the Fornell–Larcker criterion (Fornell and Larcker, 1981). The results are presented in Table 4.

**Table 4:** Comparison of square-root AVE values and inter-construct correlations.

Construct	1	2	3	4
1. Sustainability consciousness	**0.859**			
2. Environmental awareness	0.223	**0.709**		
3. Personal social responsibility	0.348	0.477	**0.861**	
4. Sustainability in sport	0.358	0.682	0.642	**0.838**

Bold diagonal values represent the square root of the Average Variance Extracted (√AVE) for each construct, whereas off-diagonal values represent the correlations among the corresponding composite scores. Construct distinctiveness was evaluated with reference to the Fornell–Larcker criterion (Fornell and Larcker, 1981).

As shown in Table 4, the square-root AVE values were 0.859 for sustainability consciousness, 0.709 for environmental awareness, 0.861 for personal social responsibility, and 0.838 for sustainability in sport. Each square-root AVE value exceeded the corresponding observed correlations among the composite scores. The highest observed correlation was between environmental awareness and sustainability in sport (*r* = 0.682), which remained below the square-root AVE values of both constructs. Similarly, the correlation between sustainability consciousness and sustainability in sport was 0.358, substantially below their respective square-root AVE values of 0.859 and 0.838. This pattern provides complementary evidence regarding the empirical distinctiveness of the four constructs. Because the off-diagonal values are based on observed composite-score correlations rather than latent factor correlations estimated within a single joint measurement model, these results are interpreted cautiously and in conjunction with the HTMT analysis reported below.

Given the theoretical relatedness and partial thematic overlap among the study constructs, and to complement the preceding comparison based on composite-score correlations, discriminant validity was additionally evaluated using the heterotrait–monotrait ratio of correlations (HTMT), which provides a more stringent assessment of construct distinctiveness.

As shown in Table 5, all HTMT values were below the conservative threshold of 0.85, ranging from 0.355 to 0.703. The highest HTMT value was observed between personal social responsibility and sustainability in sport (HTMT = 0.703), followed by environmental awareness and sustainability in sport (HTMT = 0.640). Importantly, the HTMT value between sustainability consciousness and sustainability in sport was 0.533, remaining well below the recommended threshold. Taken together, these findings provide additional evidence that the four study constructs are empirically distinguishable despite their theoretical relatedness and partial thematic overlap.

**Table 5:** Heterotrait–monotrait ratio of correlations (HTMT).

Construct	1	2	3	4
1. Sustainability consciousness	–			
2. Environmental awareness	0.355	–		
3. Personal social responsibility	0.444	0.571	–	
4. Sustainability in sport	0.533	0.640	0.703	–

HTMT, heterotrait–monotrait ratio of correlations. HTMT values below 0.85 were considered indicative of adequate discriminant validity. All pairwise HTMT values were below the conservative 0.85 threshold.

Although the study instruments are multidimensional, total scores were used in the primary correlation, regression, and serial mediation analyses because the analytical framework focused on the overall constructs rather than on dimension-specific effects. The confirmatory factor analyses supported the theoretically specified multidimensional structure of the individual study measures, while the reliability, convergent-validity, and discriminant-validity assessments provided additional evidence regarding their measurement properties. Accordingly, total scores were used as composite representations of sustainability consciousness, environmental awareness, personal social responsibility, and sustainability in sport in the subsequent construct-level analyses. This strategy allowed the hypothesized serial mediation model to remain theoretically parsimonious and consistent with the study’s primary objective of examining associations among the broader constructs rather than testing separate pathways for each subdimension. Nevertheless, the use of total scores may obscure potentially meaningful dimension-specific relationships, and future research should examine whether the observed associations differ across individual subdimensions.

### 2.7 Analysis of data

Data were analyzed using IBM SPSS Statistics version 22, while confirmatory factor analyses were conducted in IBM SPSS AMOS using maximum likelihood estimation. Univariate normality was evaluated using skewness and kurtosis coefficients, and all values were within ±2 (George and Mallery, 2010; Hair et al., 2019), supporting the use of parametric procedures. Descriptive statistics and Pearson product–moment correlations were calculated to summarize the study variables and examine their bivariate associations. Regression analyses were subsequently conducted, with multicollinearity and influential observations assessed using tolerance values, variance inflation factors (VIF), and Cook’s distance.

The hypothesized serial mediation model was tested using Hayes’ (2022) PROCESS Macro Model 6, with sustainability consciousness specified as X, environmental awareness as M1, personal social responsibility as M2, and sustainability in sport as Y. Indirect effects were evaluated using 5,000 bias-corrected bootstrap resamples with 95% confidence intervals and were considered statistically significant when the corresponding confidence interval did not include zero. PROCESS-based regression analysis was selected because the primary analytical objective was to estimate the direct and specific indirect associations among the observed composite scores within a prespecified serial mediation model. The study was therefore designed as a construct-level mediation analysis rather than as a latent-variable structural equation model. Confirmatory factor analyses were conducted separately to evaluate the measurement structure of the instruments before the construct-level analyses. Structural equation modeling represents a valid alternative approach and would allow simultaneous estimation of the measurement and structural components; however, adopting a latent SEM framework would constitute a different analytical specification from the present composite-score approach. Future studies may therefore replicate the proposed model using latent-variable SEM to examine the robustness of the observed associations while explicitly accounting for measurement error.

Given the cross-sectional design, direct and indirect effects were interpreted as statistical associations consistent with the hypothesized model rather than as evidence of causal or temporal mechanisms. Statistical significance was set at *p* < 0.05.

The skewness and kurtosis values of the study measures were examined to assess univariate normality. As presented in Table 6, all skewness and kurtosis coefficients were within the acceptable range of ±2, indicating no substantial departure from normality and supporting the use of parametric statistical analyses (George and Mallery, 2010; Hair et al., 2019).

**Table 6:** Skewness and kurtosis values of the study measures.

Scales	Skewness	Kurtosis
Sustainability consciousness	0.45	−0.34
Environmental awareness	0.36	−0.81
Personal social responsibility	−0.39	−0.35
Sustainability in sport	0.53	−0.78

## 3 Results

Table 7 presents the descriptive statistics and Pearson correlation coefficients for the study variables. Sustainability consciousness was positively associated with environmental awareness (*r* = 0.22, *p* < 0.01), personal social responsibility (*r* = 0.35, *p* < 0.01), and sustainability in sport (*r* = 0.36, *p* < 0.01). Environmental awareness was positively associated with personal social responsibility (*r* = 0.48, *p* < 0.01) and sustainability in sport (*r* = 0.68, *p* < 0.01). Furthermore, personal social responsibility was positively associated with sustainability in sport (*r* = 0.64, *p* < 0.01). Overall, all study variables were significantly and positively associated in the expected directions, providing preliminary empirical support for the hypothesized pattern of associations underlying the proposed serial mediation model.

**Table 7:** Descriptive statistics and Pearson correlation coefficients for the relationships between variables.

Scales	Min.	Max.	M ± SD	1	2	3	4
1. Sustainability consciousness	120.00	250.00	185.63 ± 27.97	1			
2. Environmental awareness	26.00	69.00	47.79 ± 8.68	0.22**	1		
3. Personal social responsibility	0.00	190.00	116.72 ± 42.16	0.35**	0.48**	1	
4. Sustainability in sport	77.00	175.00	124.82 ± 23.69	0.36**	0.68**	0.64**	1

**p < 0.01, *n* = 521.

**Figure 1:**
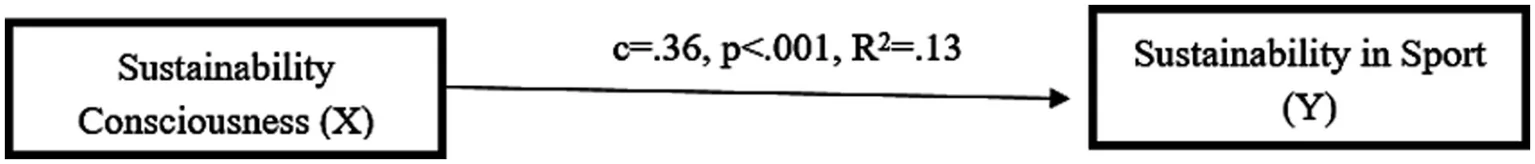
The relationship between sustainability consciousness and sustainability in sport.

Table 8 presents the results of the multiple linear regression analysis examining the association between sustainability consciousness and sustainability in sport. Sustainability consciousness was positively and significantly associated with sustainability in sport (*β* = 0.36, *t* = 8.74, *p* < 0.001). The regression model accounted for 13% of the variance in sustainability in sport (*R^2^* = 0.13), and the overall model was statistically significant (*F* = 76.40, *p* < 0.001). These findings indicate that higher levels of sustainability consciousness were statistically associated with higher levels of sustainability in sport (see Figure 1).

**Table 8:** Linear regression analysis examining the association between sustainability consciousness and sustainability in sport.

Variables									
Independent variable	Depend variable	*β*	SE	*t*	*p*	*R*	*R* ^2^	*F*	*p*
Sustainability consciousness	Sustainability in sport	0.36	0.04	8.74	< 0.001	0.36	0.13	76.40	*p* < 0.001

SE = standard error; *β* = standardized regression coefficient; *R* = multiple correlation coefficient; *R*^2^ = coefficient of determination. *N* = 521.

Table 9 presents the collinearity statistics for the predictor variables included in the regression analyses. The tolerance values for Sustainability consciousness (tolerance = 0.889), environmental awareness (tolerance = 0.766), and personal social responsibility (tolerance = 0.726) were all above the recommended threshold of 0.10, whereas the corresponding variance inflation factor (VIF) values (1.125, 1.306, and 1.378, respectively) were well below the recommended cut-off value of 10 (Hair et al., 2019). These findings indicate that no evidence of multicollinearity was detected among the predictor variables, supporting the stability of the regression estimates.

**Table 9:** Collinearity statistics of predictor variables.

Model	Collinearity Statistics	VIF
1	Tolerance	
Sustainability consciousness	0.889	1.125
Environmental awareness	0.766	1.306
Personal social responsibility	0.726	1.378

Table 10 presents the Cook’s distance statistics for the regression analyses. Cook’s distance values ranged from 0.000 to 0.064, with a mean value of 0.002 (SD = 0.004). Because the maximum Cook’s distance value was substantially below the recommended threshold of 1.00, no influential observations were identified that were likely to disproportionately affect the regression analyses (Cook, 1977; Hair et al., 2019). These findings indicate that no influential cases were detected, supporting the stability of the regression estimates.

**Table 10:** Cook’s distance statistics.

Statistic	Min.	Max.	Mean	SD	N
Cook’s Distance	0.000	0.064	0.002	0.004	521

Table 11 presents the analysis of variance (ANOVA) for the multiple linear regression model. The overall regression model was statistically significant, *F*(3, 517) = 265.188, *p* < 0.001. This finding indicates that the predictor variables, when considered simultaneously, were significantly associated with sustainability in sport and jointly explained a statistically significant proportion of its variance.

**Table 11:** Analysis of variance (ANOVA) for the multiple linear regression model.

Model	Source	Sum of Squares	df	Mean Square	*F*	*p*
1	Regression	176,824.459	3	58,941.486		
	Residual	114,910.155	517	222.263	265.188	<0.001
	Total	291,734.614	520			

*p* < 0.001.

Table 12 presents the results of the multiple linear regression analysis examining the associations among sustainability consciousness, environmental awareness, personal social responsibility, and sustainability in sport. After controlling for the other variables included in the model, sustainability consciousness remained positively and significantly associated with sustainability in sport (*B* = 0.095, *SE* = 0.025, *β* = 0.112, *t* = 3.811, *p* = 0.001). Environmental awareness was also positively and significantly associated with sustainability in sport (*B* = 1.298, *SE* = 0.086, *β* = 0.476, *t* = 15.081, *p* = 0.001), whereas personal social responsibility was positively and significantly associated with sustainability in sport (*B* = 0.213, *SE* = 0.018, *β* = 0.379, *t* = 11.694, *p* = 0.001). Among the variables included in the model, environmental awareness exhibited the largest standardized regression coefficient (*β* = 0.476), indicating the strongest statistical association with sustainability in sport after accounting for the other variables in the model.

**Table 12:** Multiple linear regression analysis of factors associated with sustainability in sport.

Model	*B*	Std. Error	*β*	*t*	*p*
1					
Sustainability consciousness	0.095	0.025	0.112	3.811	0.001
Environmental awareness	1.298	0.086	0.476	15.081	0.001
Personal social responsibility	0.213	0.018	0.379	11.694	0.001

*B* = unstandardized regression coefficient; SE = standard error; *β* = standardized regression coefficient.

**Figure 2:**
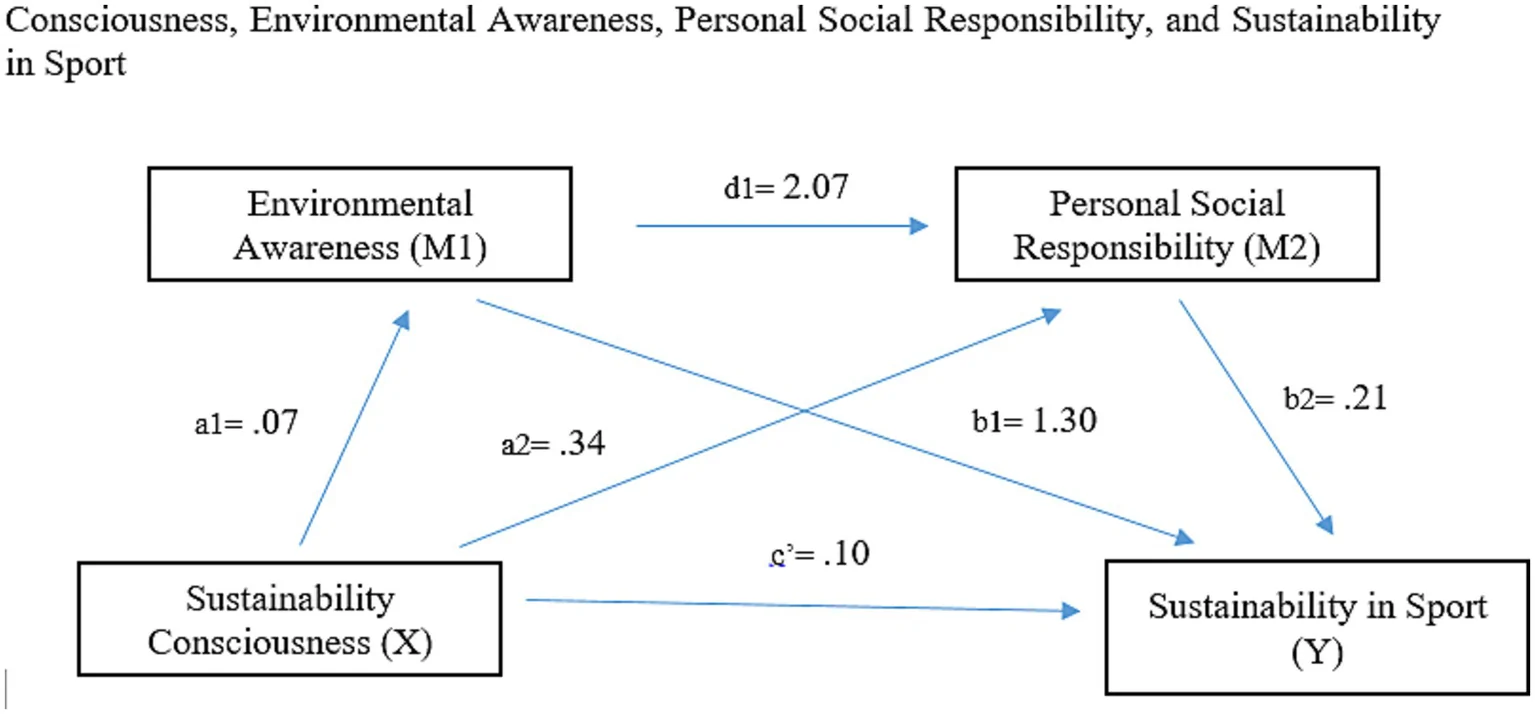
Serial mediation model examining the associations among sustainability consciousness, environmental awareness, personal social responsibility, and sustainability in sport. Path coefficients are presented in this figure; the corresponding regression coefficients and statistical results are reported in Table 13.

Table 13 presents the results of the serial mediation analysis examining the associations among sustainability consciousness, environmental awareness, personal social responsibility, and sustainability in sport. Sustainability consciousness was positively associated with environmental awareness (a₁ = 0.07, se = 0.01, *t* = 5.44, *p* < 0.001) and personal social responsibility (a₂ = 0.34, SE = 0.06, *t* = 5.79, *p* < 0.001). In addition, environmental awareness was positively associated with personal social responsibility (d₁ = 2.07, SE = 0.19, *t* = 11.05, *p* < 0.001). Environmental awareness was positively associated with sustainability in sport (b₁ = 1.30, se = 0.09, *t* = 15.08, *p* < 0.001), whereas personal social responsibility was also positively associated with sustainability in sport (b₂ = 0.21, SE = 0.02, *t* = 11.69, *p* < 0.001). After accounting for environmental awareness and personal social responsibility, the direct association between sustainability consciousness and sustainability in sport remained statistically significant (c′ = 0.10, SE = 0.03, *t* = 3.81, *p* < 0.001). The model accounted for 5% of the variance in environmental awareness (*r^2^* = 0.05), 27% of the variance in personal social responsibility (*r^2^* = 0.27), and 61% of the variance in sustainability in sport (*R*^2^ = 0.61). Taken together, these findings indicate that the observed pattern of statistical associations was consistent with the hypothesized serial mediation model (see Figure 2).

**Table 13:** Serial mediation analysis examining the associations among sustainability consciousness, environmental awareness, personal social responsibility and sustainability in sport (*N* = 521).

Outcomes	Environmental awareness (M1)				Personal social responsibility (M2)				Sustainability in Sport (Y)			
	Path	*b*	SE	*t*	Path	*b*	SE	*t*	Path	*b*	SE	*t*
Sustainability consciousness (X)	a_1_	0.07	0.01	5.44	a_2_	0.34	0.06	5.79	c’	0.10	0.03	3.81
Environmental awareness (M1)	–	–	–	–	d_1_	2.07	0.19	11.05	b_1_	1.30	0.09	15.08
Personal social responsibility (M2)	–	–	–	–	–	–	–	–	b_2_	0.21	0.02	11.69
Constant												
		*R*^2^ = 0.05				*R*^2^ = 0.27				*R*^2^ = 0.61		
		*F*(1.519) = 29.55				*F*(2,518) = 97.95				*F*(3,517) = 265.19		
		*p* < 0.001				*p* < 0.001				*p* < 0.001		

X = Sustainability Consciousness; M1 = Environmental Awareness; M2 = Personal Social Responsibility; Y = Sustainability in Sport. Unstandardized regression coefficients are reported. *p* < 0.001.

Table 14 presents the bootstrap estimates of the indirect effects of sustainability consciousness on sustainability in sport. The results indicated that the total indirect effect was statistically significant [*B* = 0.20, BootSE = 0.03, 95% CI (0.14, 0.26)]. In addition, the indirect effect through environmental awareness [Indirect Effect 1: sustainability consciousness → environmental awareness → sustainability in sport; *B* = 0.09, BootSE = 0.02, 95% CI (0.05, 0.14)], the indirect effect through personal social responsibility [Indirect Effect 2: sustainability consciousness → personal social responsibility → sustainability in sport; *B* = 0.07, BootSE = 0.02, 95% CI (0.04, 0.11)], and the serial indirect effect through environmental awareness and personal social responsibility [Indirect Effect 3: sustainability consciousness → environmental awareness → personal social responsibility → sustainability in sport; *B* = 0.03, BootSE = 0.01, 95% CI (0.02, 0.05)] were all statistically significant. Because none of the 95% bootstrap confidence intervals included zero, the findings support the statistical significance of all indirect effects.

**Table 14:** The indirect effects of sustainability consciousness on sustainability in sport (*N* = 521).

Indirect effects	*b*	SE	LLCI	ULCI
Total effect	0.20	0.03	0.14	0.26
Ind 1	0.09	0.02	0.05	0.14
Ind 2	0.07	0.02	0.04	0.11
Ind 3	0.03	0.01	0.02	0.05

## 4 Discussion

The present study offers additional evidence that is consistent with the theoretical assumptions of environmental psychology, particularly the Value-Belief-Norm (VBN) framework, by demonstrating that sustainability consciousness, environmental awareness, personal social responsibility, and sustainability in sport are systematically associated among sport sciences students. Rather than indicating that sustainability in sport is accounted for by sustainability consciousness alone, the findings suggest that environmental awareness and personal social responsibility may represent important psychological constructs associated with this sport-specific sustainability orientation. In this respect, the proposed serial mediation model contributes to the sustainability literature by providing a theoretically grounded perspective on the frequently discussed knowledge–behavior gap. Previous research has consistently shown that individuals with high levels of sustainability consciousness do not necessarily engage in environmentally responsible behaviors (Gifford, 2011; Kollmuss and Agyeman, 2002). The present findings are consistent with this perspective, suggesting that stronger sport-specific sustainability orientations are associated with higher levels of environmental awareness and personal social responsibility rather than sustainability consciousness alone.

An important measurement consideration concerns the partial content similarity between sustainability consciousness and sustainability in sport. Sustainability consciousness includes knowledge, attitudinal, and self-reported behavioral components, whereas sustainability in sport also incorporates attitudinal and practice-related aspects within a sport-specific multidimensional framework. Therefore, some degree of shared variance between the predictor and outcome is theoretically plausible. Nevertheless, the constructs differ substantially in scope and contextual referent: Sustainability consciousness represents a broad sustainability-related orientation across environmental, social, and economic domains, whereas sustainability in sport represents a domain-specific sustainability orientation embedded in the sport context. The moderate bivariate association between the two constructs (*r* = 0.36), together with the discriminant-validity evidence reported in the measurement evaluation, is consistent with related but non-equivalent constructs. However, correlation magnitude alone should not be interpreted as evidence of discriminant validity, and residual measurement overlap cannot be completely excluded. Accordingly, the magnitude of the observed associations should be interpreted with appropriate caution. This issue is especially relevant when interpreting the magnitude of associations involving sustainability in sport because part of the observed covariance may reflect similarities in item content, response format, or respondents’ general tendency to endorse sustainability-related statements. Consequently, the observed relationships may partly reflect shared measurement characteristics in addition to substantive psychological associations.

The regression analyses demonstrated that sustainability consciousness was positively and significantly associated with sustainability in sport among sport sciences students. This finding is consistent with the view that individuals with higher levels of sustainability consciousness are more likely to report stronger sustainability-related orientations within the sport context. From the perspective of environmental psychology, sustainability consciousness represents an important cognitive resource that enables individuals to recognize environmental challenges, evaluate the consequences of their actions, and incorporate sustainability principles into everyday decision-making (Steg and de Groot, 2019). Consequently, individuals with greater sustainability consciousness may be more inclined to support environmentally responsible practices related to sport participation, facility use, resource conservation, and sustainable sport management. These findings are consistent with previous research identifying sustainability consciousness as an important cognitive antecedent of sustainable behavior across diverse environmental contexts (Gericke et al., 2019; Li et al., 2019). However, the relatively modest proportion of explained variance (*R*^2^ = 0.13) suggests that sustainability consciousness alone accounts for only a limited proportion of sustainability in sport. This finding is also consistent with environmental psychology literature, which emphasizes that environmentally responsible behavior is associated with the interplay of cognitive, affective, and normative processes rather than cognitive awareness alone (Gifford, 2011; Kollmuss and Agyeman, 2002). Accordingly, although sustainability consciousness provides an important cognitive foundation, additional psychological mechanisms may be involved in understanding sustainability in sport among sport sciences students. In this context, the findings provide a theoretical rationale for examining the serial mediating roles of environmental awareness and personal social responsibility. Consistent with this interpretation, environmental awareness and personal social responsibility showed stronger bivariate associations with sustainability in sport than did sustainability consciousness, suggesting that more specific environmental appraisal and normative responsibility may be more closely associated with sport-specific sustainability orientations than a broader sustainability-related orientation alone.

The magnitude of these associations also warrants careful interpretation. Sustainability consciousness showed a relatively weak association with environmental awareness (*r* = 0.22) and moderate associations with personal social responsibility (*r* = 0.35) and sustainability in sport (*r* = 0.36). By comparison, environmental awareness and personal social responsibility were more strongly associated with sustainability in sport (*r* = 0.68 and *r* = 0.64, respectively). Although the final regression model accounted for approximately 61% of the variance in sustainability in sport (*R*^2^ = 0.61), this value should not be interpreted as evidence that the proposed psychological sequence causally explains this proportion of the outcome. Rather, it reflects the variance statistically accounted for by the predictors within the present cross-sectional, self-report dataset.

The diagnostic analyses further demonstrated that the assumptions underlying the multiple linear regression model were satisfied. The absence of problematic multicollinearity and influential observations provided support for the adequacy of the regression estimates used in the subsequent analyses (Hair et al., 2019). Furthermore, the multiple regression findings showed that sustainability consciousness, environmental awareness, and personal social responsibility were independently associated with sustainability in sport, suggesting that these constructs represent distinct yet complementary psychological dimensions. Notably, environmental awareness exhibited the largest standardized regression coefficient, followed by personal social responsibility, indicating that sustainability in sport was more strongly associated with environmental sensitivity and perceived personal responsibility than with sustainability consciousness alone. This pattern is consistent with contemporary environmental psychology, which emphasizes that sustainable behavior is associated with the interaction of cognitive, affective, and normative processes rather than knowledge alone (Steg and de Groot, 2019; Stern, 2000). Collectively, these findings are consistent with the conceptualization of environmental awareness and personal social responsibility as complementary psychological constructs associated with sustainability in sport.

The serial mediation analysis suggests that sustainability consciousness, environmental awareness, and personal social responsibility represent important psychological processes associated with sustainability in sport among sport sciences students. The findings further indicate that the association between sustainability consciousness and sustainability in sport is not fully captured by sustainability consciousness alone. Instead, this relationship appears to be associated with higher levels of environmental awareness and personal social responsibility, both of which were positively associated with sustainability in sport. This pattern is consistent with theoretical perspectives in environmental psychology suggesting that sustainability-related orientations are associated with interacting cognitive, affective, and normative processes rather than knowledge alone (Steg and de Groot, 2019). In particular, the Value-Belief-Norm (VBN) Theory proposes that awareness of environmental issues strengthens environmental beliefs, activates personal norms, and is associated with environmentally responsible behavior (Stern, 2000). Likewise, Education for Sustainable Development (ESD) emphasizes that sustainability education should extend beyond increasing knowledge to fostering environmental values, responsibility, and behavioral competencies that support sustainable action (Wals and Benavot, 2017). Collectively, these findings are consistent with the view that sustainability consciousness, environmental awareness, and personal social responsibility represent complementary psychological dimensions associated with sustainability in sport, highlighting the value of a multidimensional environmental psychology perspective for understanding sustainability in sport among sport sciences students.

Furthermore, the persistence of a statistically significant direct association between sustainability consciousness and sustainability in sport after accounting for environmental awareness and personal social responsibility indicates that both direct and indirect statistical associations remained present in the model. Thus, the observed association between sustainability consciousness and sustainability in sport was not accounted for entirely by the proposed indirect pathways.

At the same time, the results suggest that additional psychological and contextual factors may also be associated with sustainability in sport. Future research could therefore extend the proposed model by examining theoretically relevant constructs, such as environmental identity, ecological values, connectedness to nature, and pro-environmental norms, particularly using longitudinal or experimental designs to further clarify these relationships.

Alternative explanations for the observed associations should also be considered. The proposed serial ordering represents one theoretically plausible organization of the study variables, but the cross-sectional data do not establish that this ordering is uniquely supported relative to other plausible models. For example, students with stronger pre-existing sustainability orientations may simultaneously report greater environmental awareness and personal social responsibility, producing the observed pattern of associations without a sequential psychological process. Likewise, unmeasured characteristics such as environmental identity, ecological values, social norms, prior sustainability education, institutional sustainability climate, or environmental self-efficacy may contribute to multiple study variables simultaneously. Reciprocal associations are also plausible; for instance, stronger engagement with sustainability-related practices in sport may itself be associated with greater environmental awareness or perceived responsibility. Accordingly, the proposed model should be regarded as theoretically consistent with the observed data rather than as evidence that competing explanatory models have been ruled out.

The bootstrap-based serial mediation analysis indicates that the association between sustainability consciousness and sustainability in sport is better understood through multiple complementary indirect pathways rather than a single psychological mechanism. The statistically significant total indirect effect supports the presence of indirect associations involving environmental awareness and personal social responsibility. However, the specific indirect effects differed in magnitude. In particular, the stronger indirect effect through environmental awareness suggests that sustainability consciousness alone may be insufficient to account for sustainability in sport without greater sensitivity to environmental issues. This interpretation is consistent with environmental psychology, which emphasizes that awareness becomes behaviorally meaningful when individuals recognize environmental consequences and perceive environmental problems as personally relevant (Steg and de Groot, 2019; Gifford, 2011). The statistically significant indirect effect through personal social responsibility further indicates that sustainability consciousness was also associated with sustainability in sport through a stronger sense of personal responsibility toward environmental and social well-being. This finding is compatible with the Value-Belief-Norm (VBN) Theory, which proposes that environmental beliefs activate personal norms that are associated with environmentally responsible behavior (Stern, 2000). Accordingly, personal social responsibility appears to represent an important normative-motivational construct associated with sustainability in sport.

The most notable finding of the present study was the statistically significant serial indirect effect involving both environmental awareness and personal social responsibility. Although this pathway was smaller than the individual indirect effects, its statistical significance is consistent with a sequential pattern of associations among the study variables. Importantly, the magnitude of the serial indirect association was modest (*B* = 0.03) relative to the indirect associations through environmental awareness alone (*B* = 0.09) and personal social responsibility alone (*B* = 0.07). Thus, although the bootstrap confidence interval for the serial indirect effect excluded zero, its statistical significance should not be interpreted as indicating a large substantive effect. The relatively small magnitude of this pathway suggests that the proposed sequence represents one component of a broader set of psychological and contextual associations related to sustainability in sport.

Specifically, higher levels of sustainability consciousness were associated with higher environmental awareness, which was, in turn, associated with stronger personal social responsibility and higher levels of sustainability in sport. This pattern aligns with theoretical perspectives proposing that sustainability-related orientations are associated with interconnected cognitive and normative processes rather than knowledge alone (Steg and de Groot, 2019; Stern, 2000). Consequently, the findings support the usefulness of a serial mediation framework for understanding the interrelationships among sustainability consciousness, environmental awareness, personal social responsibility, and sustainability in sport. Nevertheless, the cross-sectional design imposes an important limitation on the interpretation of the serial mediation findings. Although PROCESS Model 6 permits estimation of indirect statistical associations that are consistent with the theoretically specified ordering, the simultaneous measurement of all variables does not establish temporal precedence among sustainability consciousness, environmental awareness, personal social responsibility, and sustainability in sport. Consequently, the present findings cannot demonstrate that sustainability consciousness precedes changes in environmental awareness, that environmental awareness subsequently produces changes in personal social responsibility, or that these processes ultimately lead to higher sustainability in sport. Reverse, reciprocal, or differently ordered associations remain plausible. The serial indirect effects should therefore be interpreted as statistical associations that are consistent with the proposed theoretical model rather than evidence of a causal or temporal mediation process. Longitudinal designs with temporally separated measurements and experimental or intervention-based studies are needed to evaluate the proposed ordering more rigorously.

## 5 Conclusion

The present study provides evidence of systematic associations between sustainability consciousness and sustainability in sport among sport sciences students is better understood within a multidimensional psychological framework that includes environmental awareness and personal social responsibility. The findings indicate that sustainability consciousness alone may not fully account for sustainability in sport and that environmental awareness and personal social responsibility represent complementary psychological dimensions within this relationship. By examining these constructs using a serial mediation framework, the study extends the environmental psychology literature by offering a broader perspective on the psychological factors related to sustainability in sport.

From an applied perspective, the observed associations suggest that environmental awareness and personal social responsibility may be relevant considerations when designing or evaluating sustainability-related educational initiatives in sport sciences. However, the present cross-sectional findings do not demonstrate that interventions targeting these constructs will cause improvements in sustainability in sport. Accordingly, educational programs that incorporate environmental awareness, personal responsibility, and sustainability-oriented decision-making should be regarded as theoretically informed possibilities that require evaluation through longitudinal, experimental, or intervention-based research before conclusions regarding their effectiveness can be drawn.

Several limitations should be considered when interpreting these findings. First, the cross-sectional design precludes causal inferences regarding the observed relationships. Second, the use of self-report measures may have introduced response-related biases, including social desirability and common method variance, although procedural and statistical assessments indicated that common method bias was unlikely to have substantially influenced the findings. Third, the sample consisted exclusively of sport sciences students from universities in Türkiye, which may limit the generalizability of the results to other educational, cultural, and sporting contexts. Future research should employ longitudinal and experimental designs to clarify the temporal ordering of these relationships and evaluate the effectiveness of sustainability-focused educational interventions. In addition, examining constructs such as environmental identity, connectedness to nature, ecological values, pro-environmental norms, and environmental self-efficacy may provide a more comprehensive understanding of the psychological factors associated with sustainability in sport.

## 6 Limitations

The findings of the present study should be interpreted in light of several limitations. First, the cross-sectional correlational design precludes causal inferences regarding the relationships among sustainability consciousness, environmental awareness, personal social responsibility, and sustainability in sport. Although the serial mediation model was grounded in established theoretical perspectives, including the Value-Belief-Norm Theory and Norm Activation Theory, the observed indirect effects should be interpreted as statistical associations rather than evidence of temporal or causal mechanisms. Accordingly, future longitudinal, experimental, and intervention-based research is needed to clarify the temporal ordering of these relationships and determine whether changes in sustainability consciousness are followed by changes in environmental awareness, personal social responsibility, and sustainability in sport over time.

Second, all study variables were assessed using self-report questionnaires administered within a single survey. Although procedural remedies and complementary measurement-based assessments were used to evaluate the potential influence of shared method variance, common method effects cannot be completely excluded because all measures were self-reported and collected at the same time. Self-report measures may also be susceptible to social desirability and acquiescence bias. Future studies could strengthen the validity of the findings by combining self-report instruments with behavioral indicators, observational assessments, peer or instructor evaluations, or objective measures of sustainability-related engagement within sport settings.

Third, a further limitation concerns potential measurement overlap between sustainability consciousness and sustainability in sport because both instruments contain attitudinal and self-reported behavioral elements. Although the constructs differ conceptually in scope and contextual referent and the discriminant-validity analyses supported their empirical distinctiveness, some residual shared content variance cannot be completely excluded. Future studies may therefore benefit from using more narrowly defined predictor measures and behavioral or observational indicators of sustainability-related outcomes.

In addition, although total scores were used to represent the higher-order constructs in the primary analyses, this approach may mask dimension-specific associations. Future studies should therefore examine the subdimensions separately to determine whether particular components of sustainability consciousness, environmental awareness, personal social responsibility, or sustainability in sport show differential relationships.

Fourth, participants were recruited using convenience sampling and the sample consisted exclusively of undergraduate students enrolled in Faculties of Sport Sciences at six universities in Türkiye. Consequently, the sample should not be considered statistically representative of the broader population of sport sciences students in Türkiye. Although this population represents an important group of future coaches, physical education teachers, sport managers, and recreation specialists, the non-probability sampling strategy and population-specific composition of the sample limit the generalizability of the findings to students from other academic disciplines, elite athletes, recreational sport participants, sport administrators, or populations from different cultural and educational contexts. Future studies should employ probability-based or more systematically stratified sampling strategies where feasible and include participants from broader educational, cultural, and sporting populations. Cross-cultural comparative research may also help determine whether the observed relationships are consistent across countries, educational systems, and sporting environments.

Fifth, an additional limitation concerns the potential clustering of participants within the six participating universities. Students from the same institution may share educational, organizational, and contextual characteristics that could contribute to within-university similarity. Although the distribution of participants across universities is now reported, university affiliation was not retained as a participant-level identifier in the anonymized analytical dataset, preventing retrospective estimation of intraclass correlations or cluster-adjusted effects. Consequently, the primary analyses did not explicitly account for the hierarchical structure of students nested within universities. Future multicenter studies should retain a non-identifying institutional indicator and evaluate potential clustering using appropriate multilevel or cluster-adjusted approaches when a sufficient number of institutions is available.

Finally, the study focused specifically on environmental awareness and personal social responsibility as sequential psychological mechanisms associated with the relationship between sustainability consciousness and sustainability in sport. However, sustainability in sport is a multidimensional construct that is likely to be associated with a broader range of cognitive, motivational, and contextual influences. Future research could extend the proposed framework by incorporating additional constructs from environmental psychology, such as environmental identity, connectedness to nature, personal norms, perceived behavioral control, environmental self-efficacy, ecological values, environmental concern, moral obligation, organizational sustainability climate, and social norms. Examining alternative serial or parallel mediation models that include these variables may provide a more comprehensive understanding of the psychological factors associated with sustainability in sport.

## 7 Recommendations

### 7.1 Recommendations for future research

The findings of the present study suggest several directions for future research aimed at advancing the environmental psychology literature on sustainability in sport and related sustainability orientations within sport settings. First, because the present study employed a cross-sectional correlational design, causal relationships among sustainability consciousness, environmental awareness, personal social responsibility, and sustainability in sport cannot be established. Future longitudinal, experimental, and intervention-based research is therefore needed to clarify the temporal ordering of these relationships and examine whether changes in sustainability consciousness are accompanied by subsequent changes in environmental awareness, personal social responsibility, and sustainability in sport over time.

Second, the serial mediation model was examined exclusively among undergraduate students enrolled in Faculties of Sport Sciences in Türkiye. Future research should investigate the applicability of this framework across students from other academic disciplines, competitive and recreational athletes, coaches, physical education teachers, sport managers, sport administrators, and populations from different cultural contexts. Comparative and cross-cultural studies may help determine whether the proposed relationships are consistent across different educational systems, sporting environments, and societies.

Furthermore, future studies are encouraged to extend the proposed framework by incorporating additional constructs derived from environmental psychology and sustainability research. Variables such as environmental identity, connectedness to nature, environmental concern, ecological values, personal norms, perceived behavioral control, environmental self-efficacy, organizational sustainability climate, social norms, and green organizational culture may provide a broader understanding of the psychological factors associated with Sustainability in Sport. Testing alternative serial or parallel mediation models that include these variables may further advance understanding of the relationships among sustainability consciousness, environmental awareness, personal social responsibility, and sustainability in sport.

Finally, future intervention studies should evaluate whether educational programs designed to strengthen sustainability consciousness, increase environmental awareness, and foster personal social responsibility are associated with measurable improvements in sustainability in sport. Such research would provide stronger empirical evidence regarding the effectiveness of psychologically informed sustainability education and support the development of evidence-based educational programs for future sport professionals.

### 7.2 Practical recommendations

The present findings identify environmental awareness and personal social responsibility as psychological constructs associated with sustainability in sport; however, the cross-sectional design does not establish that modifying these constructs through education will produce corresponding improvements in sustainability-related outcomes. Therefore, the following practical recommendations should be interpreted as theoretically informed considerations rather than evidence-based claims regarding intervention effectiveness. Faculties of Sport Sciences and sport organizations may consider incorporating sustainability-related content that addresses environmental awareness, personal responsibility, and sustainability-oriented decision-making, while the effectiveness of such approaches should be evaluated through appropriately designed longitudinal and experimental studies.

In addition, universities and sport organizations are encouraged to periodically assess sustainability consciousness, environmental awareness, and personal social responsibility among students and future sport professionals. Monitoring these psychological characteristics may help identify areas requiring educational support and facilitate the development of targeted sustainability initiatives that support environmentally responsible decision-making within sport settings.

#### 7.2.1 Recommendations for faculties of sport sciences and university educators

Faculties of Sport Sciences may consider integrating sustainability-related education into professional preparation alongside technical, pedagogical, and managerial competencies. Undergraduate curricula could include topics such as environmental psychology, sustainable sport management, environmental ethics, and sustainability-focused practical experiences. Such educational components may provide opportunities for students to engage with issues related to environmental awareness and personal social responsibility; however, their effects on sustainability in sport should be established through intervention-based research.

University educators may also consider active learning approaches, including project-based learning, community service activities, outdoor environmental education, interdisciplinary sustainability projects, and campus sustainability initiatives. These approaches may provide opportunities for engagement with sustainability-related issues, but the present findings do not establish that such educational experiences will directly improve sustainability in sport. Their effectiveness should therefore be evaluated empirically.

#### 7.2.2 Recommendations for sport organizations and policy makers

National sport federations, universities, municipalities, and governmental organizations responsible for sport policy are encouraged to recognize sustainability as a key component of long-term sport development. Sport organizations and policy makers may consider complementing organizational sustainability strategies with educational initiatives addressing sustainability consciousness, environmental awareness, and personal social responsibility. However, because the present study did not evaluate any intervention, these recommendations should be regarded as potential directions for practice rather than demonstrated strategies for producing behavioral change.

Furthermore, sustainability-related competencies should be systematically integrated into coach education programs, sport management curricula, and professional development initiatives. Policies that support environmentally responsible facility management, sustainable sporting events, resource conservation, waste reduction, and environmentally responsible organizational practices may help foster a sustainability-oriented culture throughout the sport sector. Strengthening both psychological competencies and organizational capacity may also support broader sustainability efforts that align with the United Nations Sustainable Development Goals, particularly SDG 4 (Quality Education), SDG 12 (Responsible Consumption and Production), and SDG 13 (Climate Action).

## Data Availability

The original contributions presented in the study are included in the article/supplementary material, further inquiries can be directed to the corresponding authors.
